# Salinity Tolerance Thresholds and Acclimation Potential in the Invasive Macrophyte *Crassula helmsii*

**DOI:** 10.1002/ece3.74496

**Published:** 2026-10-14

**Authors:** Corentin Gaudichet, Michèle Tarayre, Gabrielle Thiébaut

**Affiliations:** ^1^ UMR 6553 ECOBIO (CNRS—Université de Rennes) France

**Keywords:** invasive aquatic plant species (IAPS), salt stress, salt toxicity, salt‐tolerance

## Abstract

The salt gradient is a major driver of the composition of aquatic plant communities. The ionic composition of salt and exposure regime can influence the response of macrophytes, either positively or negatively. In European freshwaters, the New Zealand pigmyweed ( *Crassula helmsii* ) is a highly invasive macrophyte that should exhibit a small tolerance to salt, raising interest in salt‐based control strategies. Two ex‐situ experiments were conducted to evaluate the effects of salinity on *C. helmsii* . The first experiment assessed survival and morphological responses to four salinity levels (0, 15, 30 and 60 g/L) using either road or sea salts. The second experiment compared gradual versus direct exposure to elevated salinity (20 and 30 g/L). Salinity exposure reduced apical growth and biomass. No significant differences were detected between the effects of road salt and sea salt. Median effective concentration (EC50) was approximately 33 g/L. Moderate salinity stimulated branching and root production, whereas higher salinity (> 15 g/L) inhibited growth and increased mortality. Gradual exposure did not improve survival significantly compared with direct exposure. *C. helmsii* continued to grow at salinity levels up to 15 g/L, exceeding previously reported tolerance thresholds for the tested species. These findings indicate substantial tolerance to short‐term stress and delayed mortality under progressive salinisation may suggest potential acclimation ability. Eradication of *C. helmsii* would require prolonged exposure to salinity levels approaching seawater concentration that should negatively affect other freshwater organisms and ecosystem functioning. Species salinity tolerance and potential acclimation suggest an increased invasion risk of *C. helmsii* for temporarily salinised freshwater ecosystems.

## Graphical abstract

*Crassula helmsii* tolerates 15 g/L salinity for 21 days without experiencing mortality, and gradual exposure to high salt concentration may increase its salt tolerance. Therefore, shallow ponds that experience salt pulses are particularly vulnerable to this invasive species.

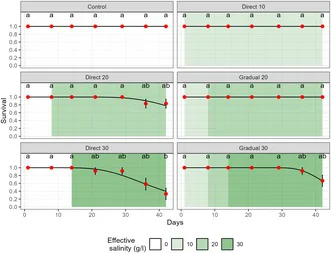

## 1 Introduction

Salinisation is a worldwide process due to anthropogenic activities and global warming (Hintz and Relyea 2019; Jeppesen et al. 2020). Mechanisms of stress response to salt are known in both plants (Avni et al. 2020; Pierik and Testerink 2014) and animals (Leprêtre et al. 2025). Most freshwater organisms experience ionic or osmotic stress when exposed to high salt concentrations, which can induce a loss of fitness or death (Moreira et al. 2023; Santagata et al. 2008). Salt treatments have been tested on clams, snails and macrophytes to control species invasiveness in ex‐situ studies (e.g., Matheson et al. 2007; Thouvenot et al. 2012; Paolucci and Thuesen 2020; Tang and Aldridge 2021). Salt‐based management of plant and animal invasiveness is widely applied, targeting either the spread or establishment phases of biological invasions.

To reduce anthropogenic translocations of invasive organisms, several decontamination processes based on salinity exposure have been implemented. For example, open‐ocean ballast water exchange has been used to prevent ships from spreading invasive species between low‐salinity ports in the USA (Santagata et al. 2009; Santagata et al. 2008), while salt‐based disinfection of fishing equipment has been implemented to prevent species transfer among inland waterbodies in New‐Zealand (Matheson et al. 2007).

Only a limited number of in situ studies have investigated salt application or seawater intrusion as control methods against invasive species, although additional cases are reported in the grey literature. For instance, seawater intrusion has been employed against invasive *Phragmites australis* (European genotype) in the USA (Gettys et al. 2009), or saltwater spraying on terrestrial *Ludwigia spp*. has been tested in France (Haury and Barloy 2018). Both trials show partial control of the species. In contrast, salinity reduction has been successfully used to eradicate the marine macroalga *Caulerpa taxifolia* from artificial saline lakes in South Australia (Walters 2009).

Seawater intrusion or salt application may represent potential management tools for invasive macrophytes in coastal or estuarine waterbodies and marshlands. Freshwater macrophytes exhibit marked interspecific variation in salinity tolerance thresholds. In their review, Moreira et al. (2023) display salinity tolerance values ranging from 0.40 to 16.65 g/L, across 30 freshwater macrophyte species, with a mean threshold of 5.25 g/L. This study indicated that a salinity level about half seawater concentration (i.e., 16.5 g/L) is highly lethal for most freshwater aquatic plants, including numerous invasive species in Europe. In addition to concentration effects, ionic composition influences toxicity in macrophytes, with NaCl being slightly more toxic than seawater (Venâncio et al. 2022), and MgCl_2_ exhibiting higher acute toxicity than seawater exposure (Coldsnow and Relyea 2021).

*Crassula helmsii* (Kirk) Cockayne is an invasive aquatic plant species within European oceanic climate area, primarily invading freshwater waterbodies and their surrounding banks. Its high biomass production and groundcover expansion result in local reduced abundance of native species and competitive exclusion (Dawson 1994; van der Loop, Tjampens, et al. 2020). The species is known for its wide ecological amplitude along the moisture gradient (Dawson 1994; Hussner 2021), as well as its tolerance to desiccation (Gaudichet et al. 2026). While, *C. helmsii* can grow in saline habitats within its native range (Dawson and Warman 1987), such behaviour has not been observed in Europe (Dean et al. 2013). Current large‐scale eradication methods, including mechanical, physical and chemical approaches, generally show limited long‐term effectiveness (van der Loop, Beringen, et al. 2020).

Given that *C. helmsii* frequently occurs in coastal or estuarine wetlands in the Atlantic area, salt‐based treatments and seawater intrusion have been proposed as potential eradication methods. Previous ex‐situ experiments reported a negative relationship between salinity and relative growth rate, concluding that 8 ppt NaCl (salinity≈8 g/L) may represent the minimum lethal concentration for the species after 31 days of exposure (Dean et al. 2013). Additionally, field experiments using seawater intrusion to locally eradicate or control the species have been reported as successful in the UK in the short term (Charlton et al. 2010; EPPO 2014). Whereas unsuccessful eradication attempts using road salt inputs in a pond have been brought to our knowledge in France (pers. comm.). These contrasting outcomes highlight uncertainties regarding lethal salinity thresholds and the role of salt composition on toxicity.

Moreover, from a practical perspective, we question the potential acclimation of *C. helmsii* to increasing salt concentration (e.g., due to evaporation or progressive salinisation). Salt acclimation has been poorly studied in freshwater macrophytes (James et al. 2003) in comparison with terrestrial plants. Some agricultural studies reported improved salt‐tolerance of *Oryza sativa* , *Zea mays* , *Brassica napus* , *Vigna unguiculata* and *Glycine max* after gradual salt exposure (Djanaguiraman et al. 2006; Pandolfi et al. 2016; Santangeli et al. 2019; Silveira et al. 2001; Umezawa et al. 2000, respectively).

Therefore, the present study investigated salinity tolerance in *C. helmsii* under ex‐situ conditions by examining the effects of salt concentration, salt type and exposure regime. We hypothesised that (i) *C. helmsii* survives at salinity levels exceeding 8 g/L, (ii) sea salt induces higher acute toxicity than road salt and (iii) progressive exposure to target salinity increases tolerance relative to direct exposure.

## 2 Materials and Methods

To test the three hypotheses, two experiments were conducted in controlled conditions in the climate chamber facility of Rennes University. The first experiment assessed the effects of salt concentration and salt type, whereas the second experiment investigated the effects of exposure regime (hereafter referred to as the exposure method) and salt concentration on salinity tolerance.

### 2.1 Plant Material

Emergent stems of *Crassula helmsii* were collected from a slightly acidic and oligo‐mesotrophic pond located in the natural area *Les Landes de Jaunouses* (Brittany, France, coordinates: 48.276 N, −1.238 E). Sampling was performed on 27 February 2025 for Experiment 1 and on 6 April 2025 for Experiment 2. Plant material was maintained under laboratory conditions prior to the start of the experiments.

For both experiments, the selected ramets consisted of 5 cm apical stem fragments lacking roots and axillary buds. The ramets were grown individually in glass pots (diameter = 60 mm, height = 40 mm) filled with saline solution (see below). The experiments were conducted in climate chambers under an 8/16 h light/dark photoperiod using LED lighting (PPFD = 320 μmol/s) at a constant temperature of 21°C.

### 2.2 Salt Material

To compare the effects of seawater intrusion and road‐salt application, we used natural coarse salt (Le Guérandais, batch n° 4,297,161,121) to mimic seawater, and for road salt (Quadrimex, norme NFP 98–180). Both salts exhibited similar ionic compositions (Table S1). Only road salt was used in Experiment 2.

Air‐dried salt was weighed using a precision balance and dissolved in water according to the treatments. Water originated from the pond where plant material had been collected. Saline solutions were renewed every ten days with fresh pond water. To limit salinity changes resulting from evaporation within the experimental units, the water level was maintained at a constant level and conductivity was monitored regularly.

### 2.3 Experimental Setup

#### 2.3.1 Experiment 1

Experiment 1 started on 4 March 2025 and lasted for 3 weeks. Four salt concentrations (0, 15, 30 and 60 g/L) and two salt types (road and sea salts) were tested. Each treatment was replicated 10 times, and plots were arranged in a randomised block design.

#### 2.3.2 Experiment 2

Experiment 2 started on 17 April 2025 and lasted 6 weeks. To assess whether salinity tolerance in *C. helmsii* may be induced through acclimation, ramets were exposed either directly or gradually to the target salt concentration. Control treatment was continuously exposed to freshwater (0 g/L). Under gradual exposure regime, ramets were initially exposed to 10 g/L NaCl during the first week, and salinity was subsequently increased by 10 g/L each week until the target concentration was reached. Under direct exposure regime, the target salinity was applied immediately (Figure 1). To standardise exposure duration among treatments, direct exposure treatments were staggered over time: the *Direct 10* treatment was initiated during Week 1, *Direct 20* during Week 2, and *Direct 30* during Week 3. Each treatment was replicated 12 times and plots were arranged in a randomised block design.

**FIGURE 1:**
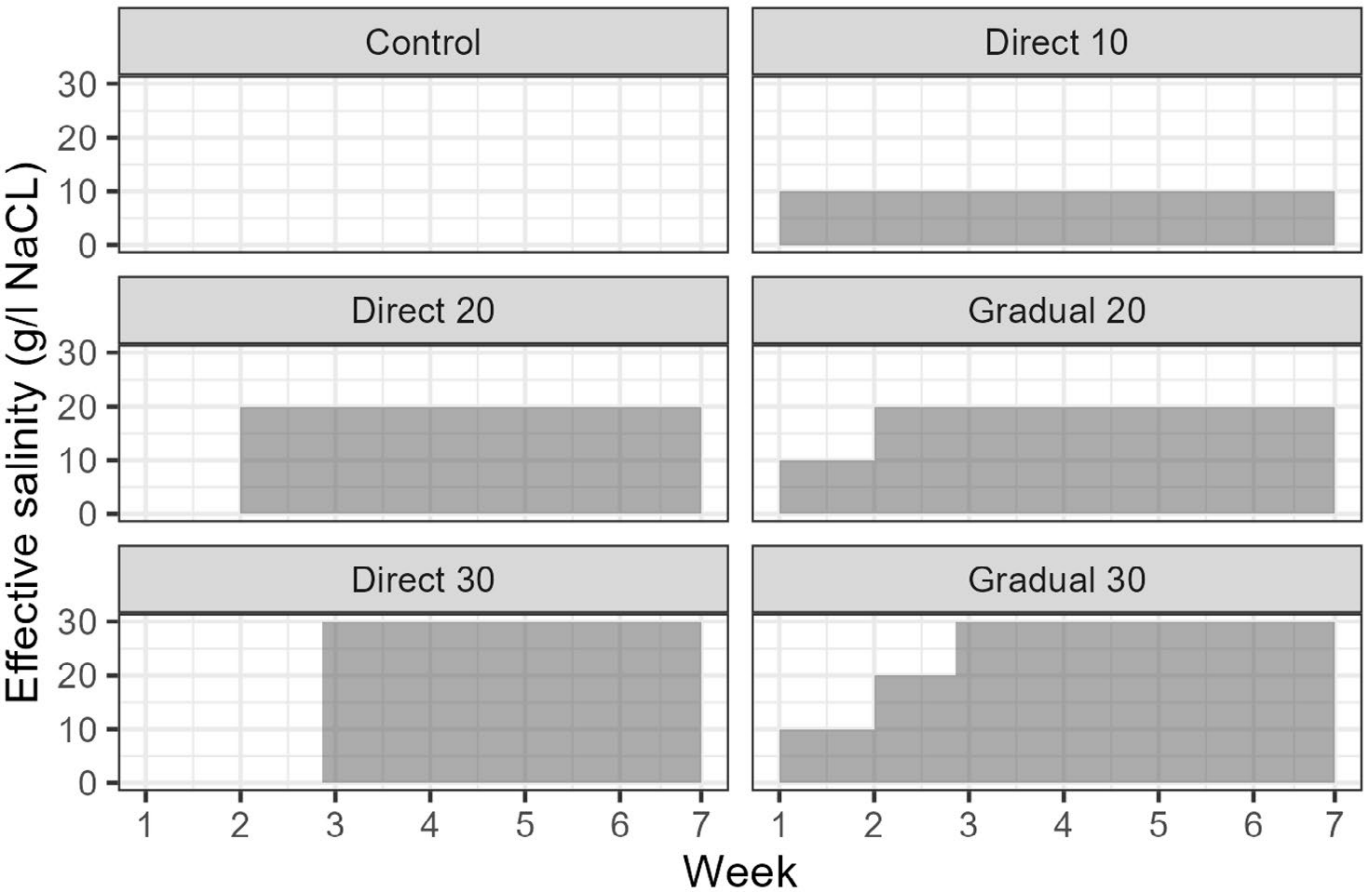
Temporal dynamics of salinity treatments applied during Experiment 2. Labels indicate treatment identity according to exposure method (direct vs. gradual) and target salinity (10, 20 or 30 g/L).

### 2.4 Trait Measurements

Ramet survival was visually assessed using a binary scale (i.e., 0 = all tissues desiccated; 1 = at least one green or greenish bud remaining).

Ramet length, ramification number and maximum branch length, as well as root number and maximum root length were counted and measured weekly. Fresh and dry weight were measured at start using a pool of 10 ramets, and individual ramets were weighted fresh and dry (Experiment 2 only) at the end of experiments. Relative growth rate (RGR, Equation 1) was calculated using length (RGR‐L) or dry weight data (RGR‐DW, Experiment 2 only). In addition, water content was calculated in Experiment 2.

(1) $$\mathrm{RGR}=\frac{\log\left(X_{\mathrm{fin}}\right)-\log\left(X_{\mathrm{ini}}\right)}{t_{\mathrm{fin}}-t_{\mathrm{ini}}}$$

Equation (1) Relative growth rate (RGR), *X*_ini_ and *X*_fin_ correspond to initial and final values (for length or dry weight), respectively, and *t*_ini_ and *t*_fin_ represent initial and final experimental dates.

### 2.5 Statistical Analysis

A loglogistic regression model (‘*drm*’ function, 2 parameters) was used to draw the concentration response curve with regard to ramets (EC_50_) between salt types was compared using a *t*‐test (‘*compParm’* function).

Based on (generalised) linear models, an ANOVA Type II procedure was performed to test the effects of treatments or time and treatments on response variables (i.e., traits). We used the Poisson error distribution (log link function) for count data and binomial error distribution (complementary loglog link function) for survival data. Models' validity was checked visually using ‘simulateResiduals’ function. When model validity was not supported, we applied an inverse normal transform procedure (INT, Equation 2) as a nonparametric alternative method (Luepsen 2018; Soloman and Sawilowsky 2009; Tsandilas and Casiez, under review). Based on estimated marginal means (*‘emmeans’* function) post hoc pairwise comparisons were performed with Bonferroni correction for *p*‐values.

(2) $$INT\left(Y\right)=\Phi^{-1}\left(\frac{\mathrm{rank}\left(Y\right)-0.5}{N}\right)$$

Equation (2) Rank‐based inverse normal transformation (INT) using the Rankit formulation, where $\Phi^{-1}$ is the standard normal quantile function and *N* is the total number of observations.

For Experiment 1, both salt concentration and salt type and their interaction were tested. Note that control group data were duplicated to maintain a balanced design. For Experiment 2, one‐way ANOVAs were performed.

Statistical analyses were run under R environment (version 4.3, R core team 2023) using car (Fox and Weisberg 2018), DHARMa (Hartig 2022), drc (Ritz et al. 2015), emmeans (Lenth 2024), lme4 (Bates et al. 2015), and multcomp (Hothorn et al. 2008) packages. Graphical layouts were produced with cowplot (Wilke 2024) and ggplot2 (Wickham 2016) packages.

## 3 Results

### 3.1 Experiment 1

Concentration response curves between road and sea salts seemed similar between salt types, with survival decreasing at concentrations equal to or over 30 g/L, and full mortality occurring at 60 g/L after 21 days of exposure (Figure 2). The median effective concentration (EC_50_) did not differ significantly between salt types (*t*_Road‐Sea_ = 0.034, *p* = 0.973).

**FIGURE 2:**
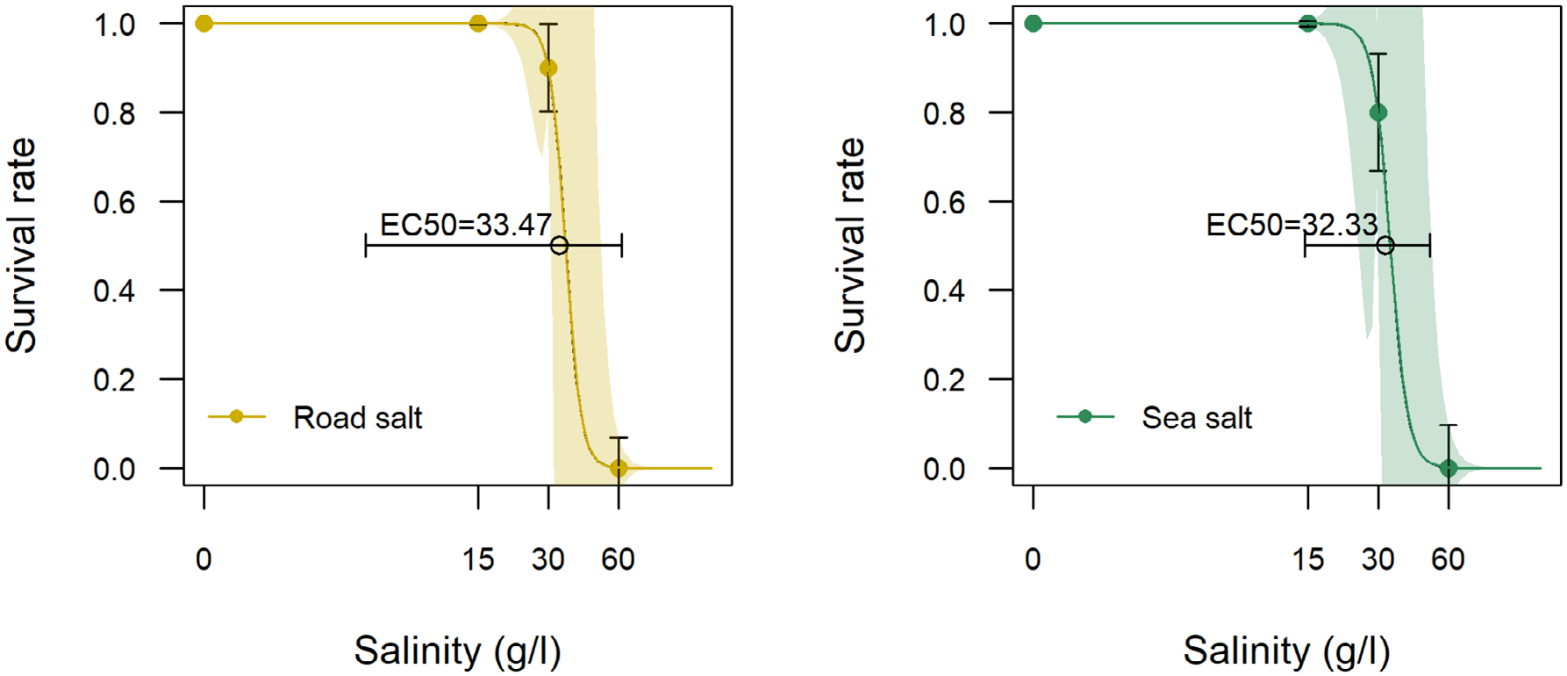
Log‐logistic regression of survival rate (mean ± SD) as a function of salt concentration for road salt (left panel) and sea salt (right panel). Open circles indicate median effective concentration (estimate ± SE).

At the end of Experiment 1, plant traits varied with salt concentration, independently of salt type (Figure 3, Table S2). For a given concentration, ramets responded similarly regardless of the salt type to which they were exposed. Ramets' relative growth rate decreased with increasing salt concentration (Figure 3A), while biomass was significantly higher in the control group than in all salt‐treated groups (Figure 3B). Exposure to a salt concentration of 15 g/L significantly increased the number of roots and ramification compared with the control and the other treatments (Figure 3C–E). There was no significant difference in ramification length among treatments (Figure 3D). Root length decreased significantly with salt concentration (Figure 3F).

**FIGURE 3:**
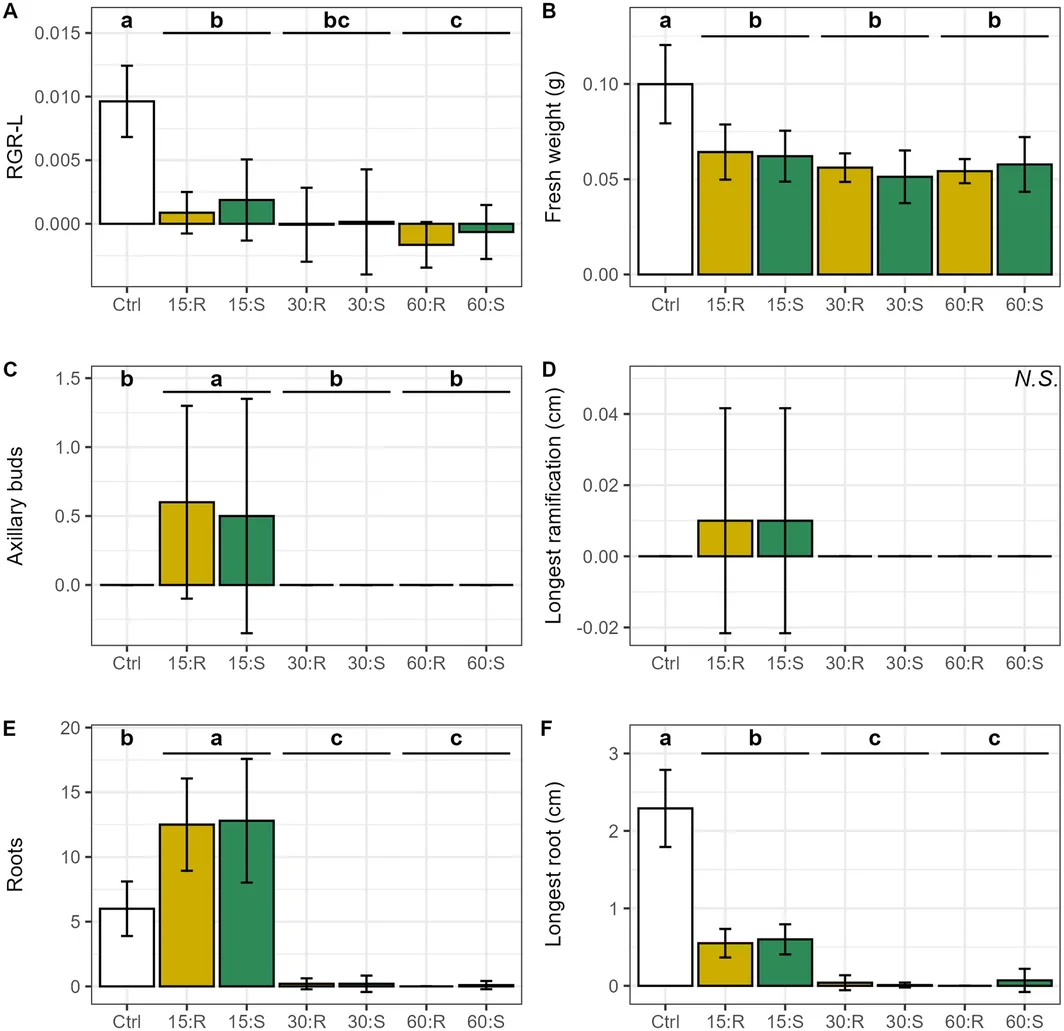
Effects of salt treatments on Crassula helmsii traits after 21 days of exposure (mean ± SD). Treatments are coded according to salinity concentration (g/L) and salt type (R = road salt; S = sea salt) and Ctrl = control. (A) relative growth rate in length (RGR‐L), (B) fresh weight, (C) number of axillary buds, (D) size of longest ramification, (E) number of roots, (F) size of the longest root. Horizontal bars indicate post hoc comparison groups. Treatments not sharing a letter are significantly different (with alpha = 0.05). N.S. indicates non‐significant differences.

### 3.2 Experiment 2

In Experiment 2, only the *Direct 30* treatment exhibited a significant difference in survival rate compared with the control treatment at the end of experiment (Figure 4, Table S3). The control, *Direct 10* and *Gradual 20* treatments did not experience any mortality. The *Direct 20* and *Gradual 30* treatments showed a decrease in survival rate from Day 36 to Day 42 with a final survival rate of 0.8 and 0.7, respectively; however, these did not differ significantly from the other treatments. In the *Direct 30* treatment, the survival rate decreased from 21 (i.e., 7 days after reaching the target concentration) until the end of the experiment, reaching a final value of 0.3, which was significantly lower than the control.

**FIGURE 4:**
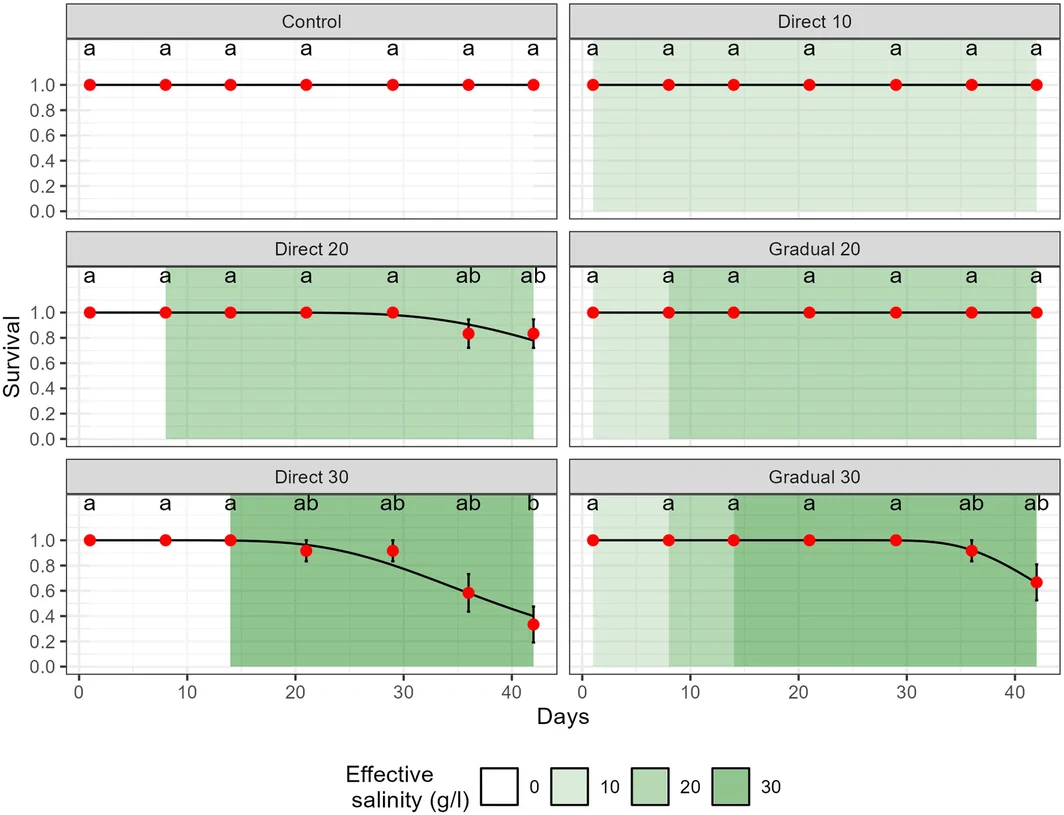
Survival dynamics of Crassula helmsii during Experiment 2 (mean ± SE). Black lines represent GLM fits (binomial family with complementary log–log link function). Treatments not sharing a letter are significantly different (with alpha = 0.05).

At the end of Experiment 2, all measured traits varied significantly among treatments, except RGR‐DW (Figure 5, Table S4). The water content decreased with concentration (Figure 5B), with *Control* mean (mean ± SD = 0.929 ± 0.009) differing from all other treatments, and *Direct 10* (0.887 ± 0.0226) having higher water content than *Direct 30* and *Gradual 30* (0.855 ± 0.025 and 0.856 ± 0.039, respectively). The mean relative RGR‐L remained positive across all treatments (Figure 5C) and was significantly higher in the control (0.003 ± 0.003) than in the *Gradual 30* treatment (0.000 ± 0.001). All treatments significantly inhibited biomass accumulation in comparison with the control treatment (Figure 5D). With regard to axillary buds production, the control (2.583 ± 1.676) and *Direct 10* (3.500 ± 1.784) treatments did not differ significantly (Figure 5E). However, all other treatments produced fewer ramifications than *Direct 10*. In addition, their ramifications were shorter than in the control or *Direct 10* treatments (Figure 5F). With regard to roots production (Figure 5G), *Direct 10* exhibit more roots (14.666 ± 5.614) than all other treatments, and *Direct 20* and *Direct 30* treatments inhibited root production in comparison with control treatment (8.583 ± 4.295). Moreover, *Direct 20* produced less roots than *Gradual 20* (5.083 ± 3.147 vs. 9.917 ± 4.999, respectively) and *Direct 30* produced less roots than *Gradual 30* (3.083 ± 1.676 vs. 7.583 ± 4.602, respectively). Root length was inhibited in the *Direct 20* treatment compared with the control (0.433 ± 0.264 cm vs. 0.942 ± 0.704 cm, respectively), whereas no other treatments significantly affected root length (Figure 5H).

**FIGURE 5:**
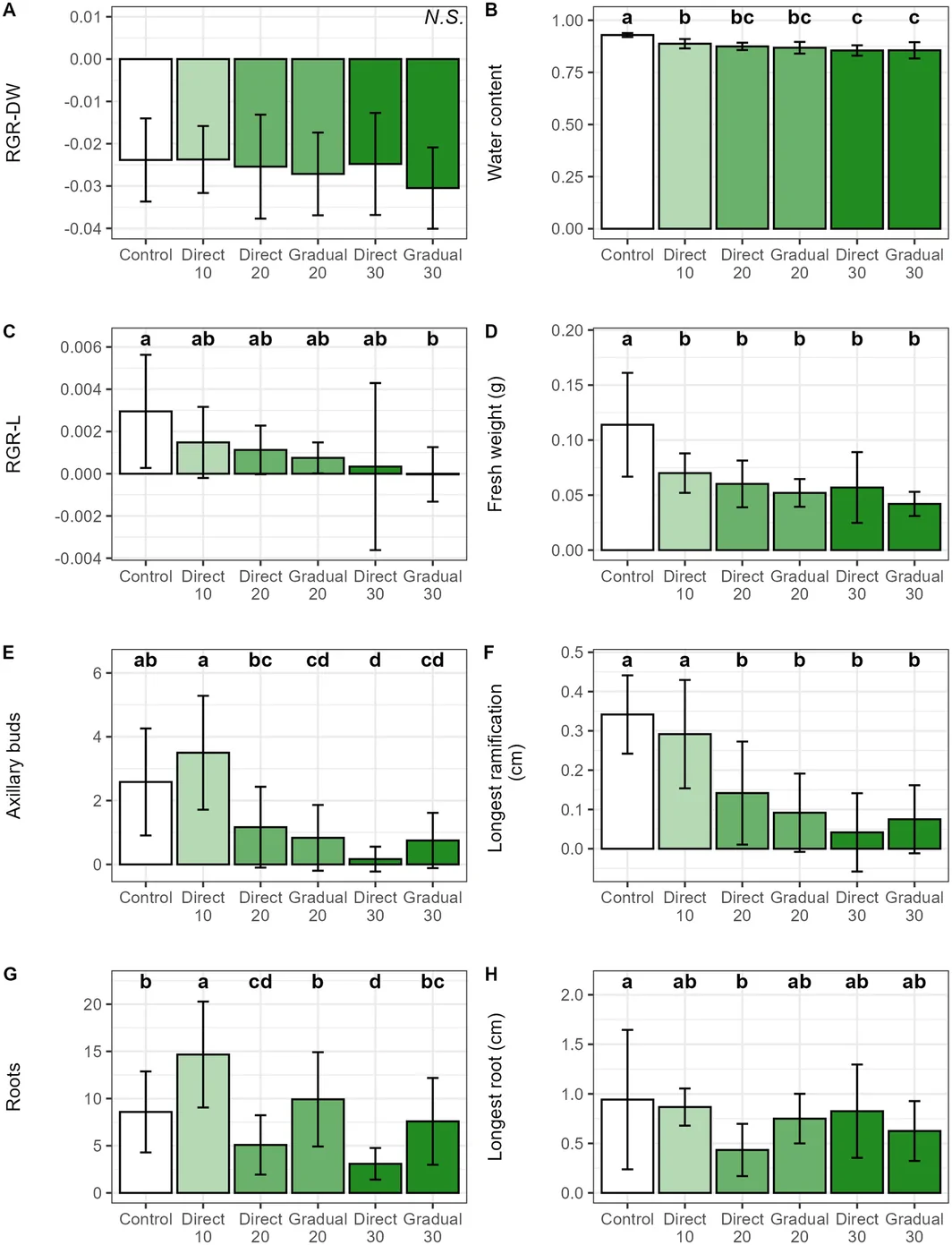
Effect of road salt exposure on *Crassula helmsii* 's traits (mean ± SD) after 41 days of exposure. Treatments are control, direct exposure or gradual exposure associated with target salt concentration (0, 10, 20 or 30 in g/L). Colour intensity indicates the target salt concentration. (A) relative growth rate in dry weight (RGR‐DW), (B) water content, (C) relative growth rate in length (RGR‐L), (D) fresh weight, (E) number of axillary buds, (F) size of longest ramification, (G) number of roots, (H) size of the longest root. Treatments not sharing a letter are significantly different (with alpha = 0.05).

## 4 Discussion

### 4.1 Salt Toxicity and Stress Response

The present study demonstrates that *Crassula helmsii* can exhibit substantial tolerance to salinity stress, with some plant mortality occurring above 15 g/L and complete mortality reached at 60 g/L after 21 days of exposure. These findings support our first hypothesis and position that *C. helmsii* is among the most salt‐tolerant freshwater invasive macrophytes currently described.

Under freshwater conditions, *C. helmsii* displayed strong apical dominance associated with rapid elongation and biomass accumulation. In contrast, exposure to moderate salinity levels (10–15 g/L) induced marked morphological changes, including reduced elongation, increased branching and altered root architecture. Such responses likely reflect a shift in plant functioning from resource acquisition towards persistence and vegetative propagation under stress conditions. Comparable overcompensation responses have been reported in other freshwater macrophytes exposed to moderate salinity stress, including *Myriophyllum aquaticum* at 1–6 g/L salinity (Thouvenot et al. 2012) and *Potamogeton perfolatius* at 2–12 g/L salinity (Twilley and Barko 1990).

At higher salinity levels (i.e., 20 and 30 g/L salinity), *C. helmsii* showed slowed apical growth and inhibited ramification and root production with lethal effects becoming apparent after 28 and 21 days of exposure, respectively. The delayed mortality observed at these concentrations should indicate that lethal effects were not caused by immediate osmotic shock but likely resulted from cumulative ionic toxicity and progressive physiological dysfunction. At the extreme salinity of 60 g/L, *Crassula helmsii* exhibited rapid decay and sinking, suggesting a hyper‐osmotic shock response.

In the present study, free‐floating *Crassula helmsii* ramets demonstrated a high salt‐tolerance, with a median effective concentration approaching the 35 g/L salinity of the seawater (Copin‐Montégut 1996; Tootoonchi and Gettys 2019). In comparisons with other freshwater macrophytes, *C. helmsii* exhibits a very high tolerance by maintaining suboptimal growth at 15 g/L salinity, a salt concentration which is lethal (or sub‐lethal) for others (Moreira et al. 2023). Among the most tolerant species, Moreira et al. (2023), reported upper salinity limits for *Lemna minor* (16.65 g/L), *Azolla caroliniana* (16.65 g/L) and *Lemna gibba* (14.61 g/L) among floating macrophytes, and for *Myriophyllum spicatum* (13.32 g/L), *Stuckenia pectinata* (12.00 g/L), *Potamogeton perfoliatus* (12.00 g/L) and *Myriophyllum aquaticum* (10.00 g/L) among submerged macrophytes (op. cit.).

A previous study of *C. helmsii* salt‐tolerance in the U.K. reported that salinity about 8 g/L was sufficient to inhibit growth and kill rooted plants after 31 days of exposure (Dean et al. 2013). Although our experimental conditions differed from those of the previous study, our results suggest a substantially higher tolerance to salt than has been reported. Our findings do not necessarily imply that *C. helmsii* can colonise permanent brackish waters (salinity > 3 g/L). Instead, they suggest that *C. helmsii* is able to endure high‐stress during temporary salinisation events (van der Loop, Beringen, et al. 2020). This distinction is ecologically important because many invaded habitats, particularly coastal wetlands and retention ponds, experience short‐term salinity pulses rather than stable brackish conditions.

### 4.2 Influence of Salt Composition

The type of salt did not significantly influence the morphological responses of *C. helmsii* . Concentration‐response curves for sea salt and road salt were highly similar and medium effective concentrations did not differ between treatments. Furthermore, no differences in morphological responses to salinity were observed between salt types, thereby refuting our second hypothesis.

It is plausible that commercial road salt used in this study has been produced from seawater, as its ionic composition was very similar to sea salt (Table S1), with both consisting primarily of sodium and chloride at 98%. However, some road salts differ in composition and contain substantial amounts of Mg^2+^ or Ca^2+^ associated with Cl^−^. For example, MgCl_2_ has been shown to exhibit acute toxicity on *Elodea canadensis* and zooplankton species compared to NaCl at equivalent concentrations (Coldsnow and Relyea 2021). Additionally, other major ions, such as SO_4_^2−^ have been demonstrated to negatively impact the growth of primary producers, whereas the presence of Ca^2+^ tends to mitigate toxic effects on macrophytes (Simmons 2012). A comparative study on *Hydrilla verticillata* and *Vallisneria americana* revealed that seawater induced less damage to these plants than table salt or laboratory‐grade NaCl solutions (Tootoonchi and Gettys 2019).

From an applied perspective, these findings suggest that both road and sea salts may serve as suitable surrogates for seawater in ecotoxicological assessments (Venâncio et al. 2019). However, when applying salts in freshwater systems, consideration of ionic composition and water hardness may be necessary to adjust chloride and sodium concentrations and achieve target salinity levels. Conversely, there remains a risk of overestimating seawater toxicity when inferring effects based on salt solutions.

### 4.3 Acclimation and Progressive Exposure to Salinity

Ramets exposed to gradual increase of salt concentration seemed to survive better at high concentration, but traits responses showed overall similar patterns between gradual and direct exposure. Therefore, evidence of an improvement in salt tolerance due to gradual exposure remains uncertain. A possible interpretation of these results is that gradual exposure delayed mortality rather than restoring optimal growth. These results thus tend to align with a similar experiment on *Eleocharis palustris* , *Panicum hemitomon* , *Sagittaria lancifolia* and *Scirpus americanus* , where final salt concentration affected macrophytes to a greater extent than did the influx rate (Howard and Mendelssohn 1999). Acclimation process has only been reported for the floating macrophyte *Azolla pinnata* (Rai and Rai 1999, in James et al. 2003), as well as with the submerged macrophyte *Vallisneria americana* (Twilley and Barko 1990).

Results highlight *C. helmsii* plasticity, which may provide an important ecological advantage in habitats subjected to fluctuating salinity conditions. Episodic salt pulses caused by road runoff, seawater intrusion, or drainage systems may therefore create environmental conditions that *C. helmsii* can tolerate temporarily, potentially facilitating persistence in coastal and anthropogenically disturbed wetlands.

## 5 Conclusion

This study demonstrated that *Crassula helmsii* possesses a high tolerance to acute salinity exposure relative to most freshwater macrophytes. Mortality occurred above 15 g/L, while complete mortality has been achieved at 60 g/L salt concentration. Salinity strongly altered growth and morphology, with moderate stress inducing compensatory branching responses and high salinity promoting growth suppression and delayed mortality.

Salt composition had limited influence on plant responses under the tested conditions; both salts had similar ionic composition, and elevated NaCl concentrations were the primary determinant of toxicity. In addition, gradual exposure seemed to increase survival under high salinity, which could suggest a potential role of the acclimation process in determining stress tolerance.

These findings, resulting from the laboratory conditions (isolated and free‐floating ramets), should be taken with caution when applying to field management. From a management perspective, salt‐based eradication of *C. helmsii* may require prolonged exposure to salinity levels close to seawater concentration. A limit of this approach could be the toxicity risks for non‐target organisms and freshwater ecosystem functioning (Moreira et al. 2023; Szklarek et al. 2022).

More broadly, the high tolerance and acclimation capacity observed in *C. helmsii* suggest that this species may remain viable under episodic salinisation scenarios associated with climate change, coastal seawater intrusion or human activities. Consequently, temporary brackish wetlands and anthropogenically salinised freshwater habitats may represent vulnerable environments for future invasion and persistence of this species.

## Author Contributions

**Corentin Gaudichet:** conceptualization (equal), formal analysis (lead), investigation (lead), methodology (equal), writing – original draft (equal), writing – review and editing (equal). **Michèle Tarayre:** conceptualization (equal), supervision (equal), writing – review and editing (equal). **Gabrielle Thiébaut:** conceptualization (equal), funding acquisition (lead), project administration (lead), writing – review and editing (equal).

## Funding

This work was supported by Office Français de la Biodiversité, AMI‐EEE 2023: M‐H‐V‐L4‐THI.

## Conflicts of Interest

The authors declare no conflicts of interest.

## Supporting information


**Table S1:** Concentration (in ppm) and relative proportion in major ions within pond water and salted solutions with both salt types. The last lines refers to seawater, as reference.
**Table S2:** Anova results for ramets traits in Experiments 1.
**Table S3:** Anova result for survival depending on days and treatments during Experiment 2.
**Table S4:** Anova results for ramets traits in Experiments 2.

## Data Availability

The data and code that support the findings of this study are available from data.indores.fr (https://doi.org/10.48579/PRO/GGHULG).
