# Supplementary material for: Salinity Tolerance Thresholds and Acclimation Potential in the Invasive Macrophyte Crassula helmsii
Source: Ecol Evol. 2026 Oct 14;16(10):e74496. doi: 10.1002/ece3.74496 (PMC13669661; doi:10.1002/ece3.74496)
Supplement: Supplementary file 1 [file ECE3-16-e74496-s001.docx]

# Supplementary Material

Table S1 : Concentration (in ppm) and relative proportion in major ions within pond water and salted solutions with both salt types. The last lines refers to seawater, as reference.

|  | **Sodium** | **Magnesium** | **Potassium** | **Calcium** | **Fluoride** | **Chloride** | **Nitrite** | **Sulphate** | **Bromide** | **Phosphate** | **Nitrate** | **Salinity** |
| --- | --- | --- | --- | --- | --- | --- | --- | --- | --- | --- | --- | --- |
| Pond water | 5.486 | 2.434 | 3.188 | 4.025 | 0.093 | 10.424 | 0.018 | 2.818 | n.a. | n.a. | 0.768 | 29.254 |
|  | 18.8% | 8.3% | 10.9% | 13.8% | 0.3% | 35.6% | 0.1% | 9.6% | n.a. | n.a. | 2.6% |  |
| Pond water + Sea salt | 745.970 | 12.019 | 6.440 | 8.263 | n.a. | 1300.932 | n.a. | 31.071 | n.a. | n.a. | n.a. | 2104.697 |
|  | 35.4% | 0.6% | 0.3% | 0.4% | n.a. | 61.8% | n.a. | 1.5% | n.a. | n.a. | n.a. |  |
| Pond water + Road salt | 844.121 | 5.049 | 16.562 | 13.866 | n.a. | 1455.706 | n.a. | 39.559 | n.a. | n.a. | n.a. | 2374.864 |
|  | 35.5% | 0.2% | 0.7% | 0.6% | n.a. | 61.3% | n.a. | 1.7% | n.a. | n.a. | n.a. |  |
| Distilled water + Sea salt | 634.622 | 7.072 | 1.895 | <LD | n.a. | 1127.196 | n.a. | 19.907 | n.a. | n.a. | n.a. | 1790.691 |
|  | 35.4% | 0.4% | 0.1% | n.a. | n.a. | 62.9% | n.a. | 1.1% | n.a. | n.a. | n.a. |  |
| Distilled water + Road salt | 735.122 | 1.014 | 5.846 | 0.864 | n.a. | 1273.136 | n.a. | 18.843 | n.a. | n.a. | n.a. | 2034.825 |
|  | 36.1% | 0.0% | 0.3% | 0.0% | n.a. | 62.6% | n.a. | 0.9% | n.a. | n.a. | n.a. |  |
| Sea water (Copin-Montégut, 1996)* | 10699.000 | 1289.000 | 383.000 | 418.000 | n.a. | 19237.000 | n.a. | 2683.000 | n.a. | n.a. | n.a. | 34709.000 |
|  | 30.8% | 3.7% | 1.1% | 1.2% | n.a. | 55.4% | n.a. | 7.7% | n.a. | n.a. | n.a. |  |

*Copin-Montégut G (1996). *Chimie de l’eau de mer*. Synthèses. Institut océanographique, Paris.

Table S2 Anova results for ramets traits in experiments 1.

|  | **Experiment 1** | | | | |
| --- | --- | --- | --- | --- | --- |
| **Response variable** | **Explanatory variable** |  |  |  |  |
| Model | lm | Sum of sq. | Df | F value | p |
| **RGR** | **Salt type** | 0.00000625 | 1 | 0.8096 | 0.371 |
|  | **Concentration** | 0.00142862 | 3 | 61.6697 | **<0.001** |
|  | **Salt type x Concentration** | 0.00000413 | 3 | 0.1784 | 0.910 |
|  | Residuals | 0.00055597 | 72 |  |  |
| Model | lm | Sum of sq. | Df | F value | p |
| **Fresh weight** | **Salt type** | 0.0000150 | 1 | 0.0693 | 0.793 |
|  | **Concentration** | 0.0277899 | 3 | 42.9090 | **<0.001** |
|  | **Salt type x Concentration** | 0.0001873 | 3 | 0.2892 | 0.833 |
|  | Residuals | 0.0155435 | 72 |  |  |
| Model | glm(poisson(link = log)) | LR Chisq | Df |  | p |
| **Ramifications** | **Salt type** | 0.091 | 1 |  | 0.763 |
|  | **Concentration** | 30.498 | 3 |  | **<0.001** |
|  | **Salt type x Concentration** | 0.000 | 3 |  | 1.000 |
| Model | lm(INT) | Sum of sq. | Df | F value | p |
| **Longuest ramification** | **Salt type** | 0.000 | 1 | 0 | 1.000 |
|  | **Concentration** | 0.775 | 3 | 2 | 0.122 |
|  | **Salt type x Concentration** | 0.000 | 3 | 0 | 1.000 |
|  | Residuals | 9.298 | 72 |  |  |
| Model | glm(poisson(link = log)) | LR Chisq | Df |  | p |
| **Roots** | **Salt type** | 0.040 | 1 |  | 0.837 |
|  | **Concentration** | 521.240 | 3 |  | **<0.001** |
|  | **Salt type x Concentration** | 1.380 | 3 |  | 0.710 |
| Model | lm(INT) | Sum of sq. | Df | F value | p |
| **Longuest root** | **Salt type** | 0.018 | 1 | 0.1831 | 0.670 |
|  | **Concentration** | 52.308 | 3 | 176.0139 | **<0.001** |
|  | **Salt type x Concentration** | 0.144 | 3 | 0.4836 | 0.695 |
|  | Residuals | 7.132 | 72 |  |  |

Table S3: Anova result for survival depending on days and treatments during experiment 2

|  | **Experiment 2** | | | |
| --- | --- | --- | --- | --- |
| **Response variable** | **Explanatory variable** | |  |  |
| Model | glm(binomial(link = cloglog)) | LR Chisq | Df | p |
| **Survival** | **Day** | 58.192 | 6 | **<0.001** |
|  | **Treatment** | 51.535 | 5 | **<0.001** |
|  | **Day x Treatment** | 1.578 | 30 | 1 |

Table S4: Anova results for ramets traits in experiments 2.

|  | **Experiment 2** | | | | |
| --- | --- | --- | --- | --- | --- |
| **Response variable** | **Explanatory variable** |  |  |  |  |
| Model | lm | Sum of squares | Df | F value | p |
| **RGR-DW** | **Treatment** | 0.0003956 | 5 | 0.7377 | 0.5979 |
|  | Residuals | 0.0070782 | 66 |  |  |
| Model | lm | Sum of squares | Df | F value | p |
| **Water Content** | **Treatment** | 0.04593 | 5 | 14.415 | **<0.001** |
|  | Residuals | 0.04206 | 66 |  |  |
| Model | lm | Sum of squares | Df | F value | p |
| **RGR-L** | **Treatment** | 0.00006673 | 5 | 2.7418 | **0.026** |
|  | Residuals | 0.00032128 | 66 |  |  |
| Model | lm | Sum of squares | Df | F value | p |
| **Fresh weight** | **Treatment** | 0.038405 | 5 | 10.694 | **<0.001** |
|  | Residuals | 0.047404 | 66 |  |  |
| Model | glm(poisson(link = log)) | LR Chisq | Df |  | p |
| **Ramifications** | **Treatment** | 64.819 | 5 |  | **<0.001** |
| Model | lm | Sum of squares | Df | F value | p |
| **Longuest ramification** | **Treatment** | 0.91778 | 5 | 14.987 | **<0.001** |
|  | Residuals | 0.80833 | 66 |  |  |
| Model | glm(poisson(link = log)) | LR Chisq | Df |  | p |
| **Roots** | **Treatment** | 121.15 | 5 |  | **<0.001** |
| Model | lm(INT) | Sum of squares | Df | F value | p |
| **Longuest root** | **Treatment** | 2.0557 | 5 | 2.5271 | **0.037** |
|  | Residuals | 10.7375 | 66 |  |  |
